# Lung Cancer Survival with Current Therapies and New Targeted Treatments: A Comprehensive Update from the Srinagarind Hospital-Based Cancer Registry from (2013 to 2017)

**DOI:** 10.31557/APJCP.2021.22.8.2501

**Published:** 2021-08

**Authors:** Wachiraporn Musika, Supot Kamsa-Ard, Chananya Jirapornkul, Chalongpon Santong, Anakapong Phunmanee

**Affiliations:** 1 *Bueng Khong Long Hospital, Bueng Khong Long District, Bueng Kan Province, Thailand. *; 2 *Department of Epidemiology and Biostatistics, Faculty of Public Health, Khon Kaen University, Khon Kaen, Thailand. *; 3 *ASEAN Cancer Epidemiology and Prevention Research Group, Khon Kaen University, Khon Kaen, Thailand. *; 4 *Cancer Unit, Srinagarind Hospital, Faculty of Medicine, Khon Kaen University, Khon Kaen, Thailand. *; 5 *Department of Medicine, Faculty of Medicine, Khon Kaen University, Khon Kaen, Thailand. *

**Keywords:** Lung cancer, survival, cancer registry, targeted therapy

## Abstract

**Background:**

Lung cancer (LC) is a common malignancy and leading cause of cancer death worldwide and in Thailand. An update on LC survival factors after diagnosis at Srinagarind Hospital is needed.

**Methods:**

We conducted a retrospective cohort study, and the data were sourced from the Srinagarind Hospital-Based Cancer Registry. All LC cases were diagnosed between January 1, 2013, and December 31, 2017, and followed up until November 30, 2019. Cases of LC (ICD-O-3) numbered 2,149, but only those with coding C34.0-C34.9 were included. The survival rate was estimated using Kaplan-Meier, while the Log-rank test was used to estimate survival. Hazard ratios (HRs) and 95% confidence intervals (CIs) were estimated using Cox proportional hazard regression models.

**Results:**

The 2,149 patients had a total follow-up of 269.6 person-years. Overall, 1,867 patients died during the study, for a corresponding case-fatality mortality rate of 86.0 per 100 person-years. The respective 1-, 3-, and 5-year survival rate was 31.2 % (95% CI; 29.21 to 33.15%), 12.9 % (95%CI: 11.49 to 14.45), and 10.2% (95%CI: 8.74 to 11.70). After patient diagnosis, the median survival time was 0.46 years (5.51 months) (95% CI: 0.42 to 0.50). Targeted therapy was associated with longer survival than non-targeted therapy (p-value < 0.001). After adjusting for sex, TNM stage, and histologic type, multivariable analysis of the entire cohort identified chemotherapy as an independent predictor of improved survival (adjusted HR= 0.48; 95% CI: 0.42 to 0.55; P < 0.001), and that sex, TNM stage, and histologic type were associated with survival.

**Conclusion:**

The study confirmed that sex, stage of disease, histology, and chemotherapy are associated with survival of LC. Primary prevention and screening for early detection improve survival. Further investigations into factors affecting survival of LC in Northeast Thailand should focus on targeted therapy.

## Introduction

Lung cancer (LC) is a malignant tumor with the highest global morbidity and mortality of all cancers. In 2018, the number of new cases was 2,093,876 (11.6% of all cancers), while the number of deaths was 1,761,007 (18.4% of all cancers) among both sexes for all ages (Bray et al., 2017). The respective age-standardized rate (ASR) for LC in males and females is 31.5 and 14.6 per 100,000. The respective age-standardized incidence and mortality rate for both sexes is 22.5 and 18.6 per 100,000 (International Agency for Research on Cancer, 2019). 

For Thais, the ASR for LC is between 20.6 and 27.1 per 100,000 in males and between 9.3 and 11.9 per 100,000 in females. In Khon Kaen province, Thailand, the ASR is between 18.0 and 21.1 per 100,000 in males and between 5.9 and 7.6 per 100,000 in females. The most common histological type is non-small cell lung cancer (NSCLC) (90.0%), followed by small cell lung cancer (SCLC) (10.0%) (Sriplung et al., 2003; Khuhaprema et al., 2007; Khuhaprema et al., 2010; Khuhaprema et al., 2012). 

The incidence trend for LC in Khon Kaen province, Thailand, has modestly increased over the last 20 years. By 2030, the incidence rate is predicted to increase among females but to decline among males (Santong et al., 2018). Prevention is a long-term strategy, and not all cancers can be prevented (Schottenfeld and Fraumeni, 2006); nevertheless, implementation of national and regional preventative programming has been uneven. In order to reduce LC mortality, a reduction of LC incidence and an improvement of LC survival are essential (Allemani et al., 2018). The first-ever population-based cancer survival data (1985-1992) for Khon Kaen, Thailand, were published in 1995. The study revealed that the most common cancers in the province were liver (5-year relative survival rate 9.2%), cervix (60.1%), lung (15.4%), breast (48.1%), and large bowel (41.9%) (Sriamporn et al., 1995). A later study on Cancer Survival in Khon Kaen, Thailand, revealed that the 5-year survival between 1993 and 1997 was highest for localized disease, followed by regional and distant metastatic categories. Trends in the 5-year relative survival between 1993 and 1997 vs. 1985 and 1992 showed a marked increase for cancers of the rectum, breast, ovary, Hodgkin and non-Hodgkin lymphomas, and a decrease for cancers of the lip and larynx (Suwanrungruang et al., 2011). Previous studies showed the prognostic factors in non-small cell LC patients include stage of disease, performance status (León-Atance et al., 2011), weight loss, male vs. female, age, smoking status, smoking history, quality of life, marital status, diagnosed with depression, and genetic mutations (Jazieh et al., 2000; Brundage et al., 2002). 

The prognostic factors and survival rate for LC have not been updated recently for the tertiary hospitals in northeastern Thailand where cancer patients are treated. The current research thus aimed to determine the factors affecting the survival of LC patients after diagnosis at Srinagarind Hospital.

## Materials and Methods

*Cancer Registries and Case Ascertainment*

*Khon Kean Cancer Registry, KKCR*

The Khon Kaen Cancer Registry (KKCR) was established in 1984 at the Faculty of Medicine and Srinagarind Hospital, Khon Kaen University, Khon Kaen, Thailand. It comprises both hospital and population-based registrations. The KKCR contains data on 1.7 million patients comprising all cancer sites as per the International Agency for Research on Cancer (IARC) guidelines (Esteban et al., 1995). 

*Case definitions*

The database was retrieved for all patients with LC tumors treated at Srinagarind Hospital, Faculty of Medicine, Khon Kaen University between January 1, 2013, and December 31, 2017. Diagnoses were obtained using the International Classification of Diseases for Oncology, 3rd edition (ICD-O-3). LC is an ICD-O-3 diagnosis and only includes coding C34.0-C34.9 (World Health Organization, 2013).

*Statistical methods*

*Descriptive epidemiology of study patients*

The characteristics of the patients were summarized using descriptive statistics. Means and standard deviations, medians, and their ranges (minima and maxima) were used for continuous variables, and frequency counts and percentages were used for categorical variables.

*Survival analyses*

Survival analyses excluded cases if their basis of diagnosis was Death Certificate Only (DCO) or unknown, if they did not contain any follow-up information, or had an unknown vital status. Survival was determined by calculating the follow-up time from diagnosis to each patient’s last known vital status. The status was obtained by linking records between the Mortality Registry of Thailand (National Health Office, 2017) and the National Statistical Office (National Statistical Office Thailand, 2017, updated to December 31, 2016).

The observed survival (OS) analysis was estimated using the Kaplan-Meier survival curve, and the log-rank test was used for between-group comparisons. Multivariable analysis was performed using Cox proportional hazards regression (Kleinbaum and Klein, 2005). All test statistics were two-sided, and a p-value of < 0.05 was considered statistically significant.

*Data processing*

Data were recorded using the CanReg 5 software provided by the International Association of Cancer Registries (IARC) (IARC, 2019). The verification was performed with necessary corrections, including logic, range, and internal consistency, which were checked using statistical software. All analyses were performed using Stata release 10.0 (StataCorp LLC, College Station, TX, USA). (Stata Corp, 2007) 

*Ethical considerations*

This project was reviewed and approved by the Human Research and Ethics Committee of Khon Kaen University (HE631214). 

**Table 1:** Characteristics of Study Participants Diagnosed at Srinagarind Hospital between 2013 and 2017

Characteristic	Number (n = 2,149)	Percentage (%)
Sex		
Male	1,420	66.1
Female	729	33.9
Age at diagnosis (years)		
20-29	14	0.7
30-39	47	2.2
40-49	205	9.5
50-59	571	26.6
60-69	718	33.4
70-79	485	22.6
>80	109	5.1
Mean (standard deviation)	62.4 (11.3)	
Median (minimum: maximum)	63.0 (20: 91)	
Marital status		
Single	59	2.8
Married	2041	95.1
Monk	42	2
Not specify	7	0.3
Year of Diagnosis		
2013	489	22.8
2014	497	23.1
2015	385	17.9
2016	414	19.3
2017	364	16.9
Basis of diagnosis		
History & Physical exam	5	0.2
Ultrasound (Endoscopy & Radiology)	602	28
Surgery & Autopsy (no histol.)	6	0.3
Specific Biochem/ Immuno. test	2	0.1
Cytology or Hematology	195	9.1
Histology of Metastasis	24	1.1
Histology of Primary	1315	61.2
Subtype		
Main bronchus	51	2.4
Upper lobe, lung	818	38.1
Middle lobe, lung	82	3.8
Lower lobe, lung	466	21.7
Overlapping lesion of lung	35	1.6
Lung, NOS	697	32.4
Histology		
Squamous cell carcinoma	156	7.3
Adenocarcinoma	930	43.3
Small cell carcinoma	43	2
Large cell carcinoma	160	7.5
Other specified carcinoma	34	1.6
Sarcoma	8	0.4
Non-small cell carcinoma	128	6
Other specified malignant neoplasm	9	0.4
Unspecified malignant neoplasm	681	31.7
Characteristic	Number (n = 2,149)	Percentage (%)
Histology grading		
Well differentiated	50	2.3
Moderately differentiated	56	2.6
Poorly differentiated	196	9.1
Undifferentiated	32	1.5
Not known	1815	84.5
Laterality		
Right	689	32.1
Left	541	25.2
Bilateral	34	1.6
Unknown	885	41.2
Stage of disease		
Stage I	35	1.6
Stage II	49	2.3
Stage III	356	16.6
Stage IV	1150	53.5
Unknown	559	26
Metastasis		
Lymp node metastasis	70	3.3
Bone metastasis	288	13.4
Liver metastasis	79	3.7
Treatment		
Surgery	140	6.5
Radiation	393	18.3
Chemotherapy	669	31.1
Targeted therapy	37	1.7
Supportive care	910	42.4

**Table 2:** The 1-, 3-, 5-Year Survival Rate between 2013 and 2017 for LC after Diagnosis, by Cell Type and Chemotherapy Treatment at Srinagarind Hospital

Variable	Number	Median time (95%CI)	1-year Survival rate (95%CI)	3-year Survival rate (95% CI)	5-year Survival rate (95% CI)
Cell type					
SCLC	43	0.58 (0.39 - 0.76)	27.9 (15.57- 41.65)	11.63 (4.26– 23.06)	NA
NSCLC	1374	0.60 (0.53-0.66)	37.8 (35.20- 40.34)	15.14 (13.21- 17.19)	11.9 (10.01 - 14.01)
SCLC with Chemotherapy					
Yes	22	0.60 (0.32- 0.88)	27.3 (11.12- 46.37)	NA	NA
No	21	0.28 (0.03-0.53)	28.6 (11.66- 48.18)	14.3 (3.57-32.12)	NA
NSCLC with Chemotherapy					
Yes	592	1.07 (0.97-1.17)	53.5 (49.35- 57.40)	17.9 (14.75- 21.28)	11.8 (8.72- 15.34)
No	782	0.33 (0.30-0.37)	25.8 (22.79- 28.96)	13.1 (10.78- 15.68)	11.9 (9.52- 14.46)

SCLC, small cell lung cancer; NSCLC, non-small cell lung cancer; NA,Not applicable

**Figure 1:**
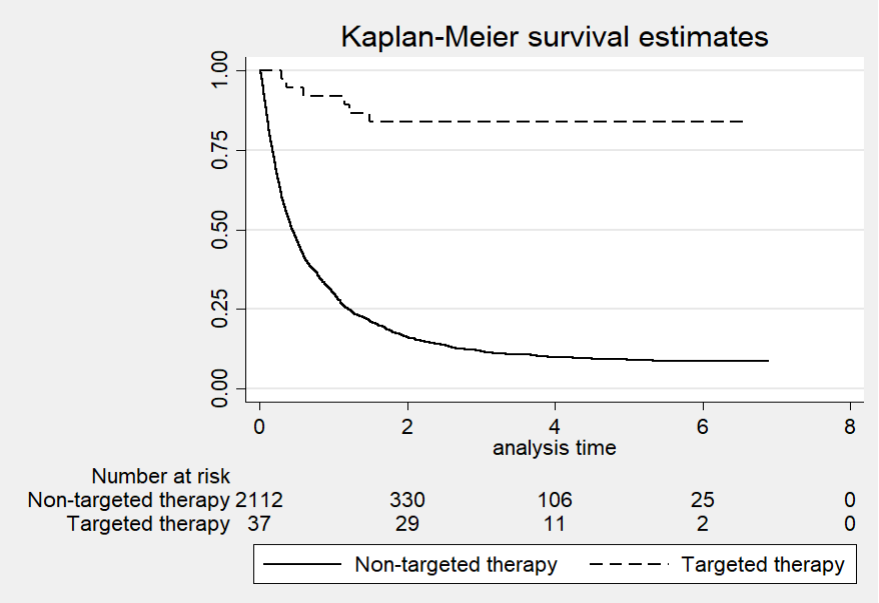
Overall Survival Based on Treatment of Patients (Targeted Therapy and Non-Targeted Therapy)

**Table 3:** Multivariable Analysis between 2013 and 2017 for Overall Survival of the Entire LC Cohort after Diagnosis at Srinagarind Hospital

Variable	Crude HR (95%CI)	Adjusted HR (95%CI)	p-value^1^
1. Sex			< 0.001
Male	1	1	
Female	0.74 (0.67 to 0.82)	0.78 (0.68 to 0.89)	
2. TNM stage			< 0.001
Stage I and II	1	1	
Stage III	3.76 (2.64 to 5.37)	6.35 (4.05 to 9.95)	
Stage IV	5.66 (4.01 to 7.98)	8.32 (5.36 to 12.90)	
3. Histologic type			< 0.001
Squamous cell carcinoma	1	1	
Adenocarcinoma	0.76 (0.63 to 0.91)	0.78 (0.63 to 0.96)	
Small cell carcinoma	0.98 (0.69 to 1.41)	0.79 (0.50 to 1.22)	
Large cell carcinoma	1.08 (0.88 to 1.32)	1.01 (0.80 to 1.27)	
4. Chemotherapy			< 0.001
No	1	1	
Yes	0.55 (0.49–0.60 )	0.48 (0.42–0.55)	

^1^p-value from partial likelihood ratio test, Adjusted HR, adjusting for Sex;TNM stage, Histologic type, and Chemotherapy

## Results

*Descriptive epidemiology and Data quality*

Between 2013 and 2017, 2,149 cases of LC were recorded in the Srinagarind Hospital-Based Cancer Registry database. Male LC patients outnumbered female LC patients. The age at diagnosis trended to be late middle-aged (mean, 62.4 years; standard deviation, 11.3; median, 63.0 years; Min: Max; 20: 91). Most were “married” (n=2,041; 95.1%). As for the year of diagnosis, the most numerous was in 2014 (n=497; 23.1%), while the least was in 2017 (n=364; 16.9%).

The basis of diagnosis was endoscopic and radiologic evidence vs. morphological verification (n=1,534; 71.4%) (i.e., based on either cytological or histological examination of tissue from the primary site, %MV). Based on the subtype of cancer, the highest was in the upper lobe (n=818; 38.1%), while the lowest was overlapping lesions of the lung (n=35; 1.6%). The most common histological grading was adenocarcinoma (n=930; 43.3%), while the highest was “unknown grading” (n=1,815; 84.5%).

The most common stage of diseases were ‘Stage IV’ (n=1,150; 53.5% and “Stage I” (n=35; 1.6%). A histological grading was commonly lacking (males; n=9,199, 97.6%; females; n=4,282, 97.9%). Tumour laterality was often unknown or undefined (n=885, 41.2%). Metastatic lung cancer was bone metastasis (n=288, 13.4%). For treatment, only targeted therapy was offered to 37 patients (1.7%) while supportive care was offered to 1,198 (55.8%) (Table 1).

*Survival rate of LC after diagnosis*

*Mortality rate and Median survival time*

The 2,149 patients had a total follow-up of 269.6 person-years. Overall, 1,867 patients died during the study, corresponding to a mortality rate (case-fatality) of 86.0 per 100 person-years. The respective 1-, 3-, and 5-year survival rate was 31.2 % (95% confidence interval; 29.21 to 33.15%), 12.9 % (95%CI: 11.49 to 14.45), and 10.2% (95%CI: 8.74 to 11.70). After diagnosis, the median survival time was 0.46 years (5.51 months) (95% CI: 0.42-0.50).

Based on the type of cancer, the respective median overall survival (OS) and 3-year OS rates for patients with small-cell lung cancer (SCLC) and non-small cell lung cancer (NSCLC) was 0.58 year (95%CI: 0.39 - 0.76) and 11.6% (95%CI: 4.26 to 23.06) vs. 0.60 years (95%CI: 0.53 to 0.66) and 15.1% (95%CI: 13.21 to 17.19). As for SCLC, the respective 1-year OS rate for patients treated with chemotherapy vs. non-chemotherapy was not significantly different. Meanwhile, NSCLC patients treated with chemotherapy over non-chemotherapy were associated with longer survival (Table 2).

*Effect of targeted therapy on survival*

Treatment of patients with targeted over against non-targeted therapy was associated with longer survival (p-value < 0.001). The respective 1-, 3-, and 5-year OS rate for patients treated with targeted therapy vs. non-targeted therapy was 91.9% (95%CI: 76.93 to 97.31), 83.8% (95%CI: 67.42 to 92.37), and 83.8% (95%CI: 67.42 to 92.37). Meanwhile, the respective 1-, 3-, and 5-year OS rate for patients receiving non-targeted therapy was 30.1% (95%CI: 28.14 to 32.07), 11.8% (95%CI: 10.38 to 13.25), and 9.0% (95%CI: 7.69 to 10.52) (Figure 1).

*Survival and multivariable Cox regression analyses*

After adjusting for sex, TNM stage, and histologic type, multivariable analysis of the entire cohort identified chemotherapy as an independent predictor of improved survival (adjusted HR= 0.48; 95% CI: 0.42 to 0.55; P < 0.001), and that sex, TNM stage, and histologic type were associated with survival (Table 3).

## Discussion

The current study investigated the factors affecting the survival of LC patients after diagnosis at Srinagarind Hospital between 2013 and 2017. We describe these issues in detail for each topic.

*Overall Survival of LC patients*

The current study showed that the respective 1-, 3-, and 5-year survival rate was 31.2% (95% CI: 29.21 to 33.15%), 12.9 % (95%CI: 11.49 to 14.45), and 10.2% (95%CI: 8.74 to 11.70). As for the five-year net survival (%) estimates, the survival rate for LC from CONCORD was 10.2%. The rate is below 10.0% in Thailand, Brazil, Bulgaria, and India (Allemani et al., 2018). The population-based data from the SEER registries in the USA shows that the 5-year relative survival for LC was 18.4% between 2005 and 2011, compared to 12.2% between 1975 and 1977 when SEER record-keeping began (SEER, 2015 ). 

*Survival of LC by targeted therapy*

Despite some improvement in survival among patients treated with targeted therapies, LC remains one of the most fatal cancers. Based on available data, the current study showed that the respective 1-, 3-, and 5-year survival rate between targeted therapy and non-targeted therapy was 91.9% (95% CI: 76.93 to 97.31 vs. 30.1% (95% CI: 28.14 to 32.07%), 83.8% (95% CI: 67.42 to 92.37) vs. 11.8% (95% CI: 10.37 to 13.25 %), and 83.8% (95% CI: 67.42 to 92.37) vs. 9.0 % (95% CI: 7.69 to 10.52%). The result is consistent with previous studies. Survival outcomes in patients with advanced non-small-cell lung cancer - treated with erlotinib - expanded access to the program data from Belgium (the TRUST study). Overall the respective survival rates at 1, 2, and 3 years was 26.4%, 10.9%, and 6.4% (Van Meerbeeck et al., 2014). Survival of patients with advanced NSCLC treated with first-generation EGFR-TKIs at a cancer hospital in Thailand (between 2011 and 2016). The respective overall observed survival rates of patients receiving EGFR-TKIs as first-line or maintenance (n=18), second-line (n=18), and third-line or more (n=14) was 15.9% (95%CI: 10.26 to 21.46), 10.9% (95%CI: 0.00 to 28.29), and 20.2% (95%CI: 6.26 to 34.21) (Sukauichai et al., 2018 ). 

The respective 1- and 2-year observed survival rate of patients receiving Gefitinib was 85.0% and 57.9%. For patients receiving carboplatin-paclitaxel for chemo-naïve non-small cell lung cancer with sensitive EGFR gene mutations, the respective 1- and 2-year survival NEJ002 (CBDCA/PTX) was 86.8% and 53.7% (Inoue et al., 2013). 

*Survival factors of LC patients*

*Sex*

In Khon Kaen, Thailand, over the last 20 years, the trend incidence for LC has increased for both sexes. (Santong et al., 2018) Male/female is the one factor that may influence survival. Previous studies have reported that for most cancers being female provides a survival benefit over being male (Micheli et al., 2009; Cook et al., 2011), although females have a higher risk of death from bladder cancer (Zaitsu et al., 2015).

The current statistical work-up shows that male/female is a significant risk factor for LC patient survival. After adjusting all the variables in the model, the mortality risk of being female was 0.78–fold compared to male patients (adjusted HR=0.78, 95%CI: 0.68 to 0.89). The result is consistent with previous studies in the USA, confirming a female survival benefit over male for LC irrespective of histologic type. [30] Since then, other studies on male vs. female difference vis-à-vis LC survival have confirmed the trend (Sagerup et al., 2011; Li et al., 2019). 

A similar survival benefit trend accrued to females after adjusting for years since cessation and smoking dose. The hazard ratio (HR) for LC mortality-comparing the association with smoking in women to that in men was 0.90 (adjusted HR=0.90, 95%CI: 0.80 to 0.90) for current smokers and 0.9 (adjusted HR=0.90, 95%CI: 0.90 to 1.00) for former smokers (Freedman et al., 2008). 

*TNM stage*

The current study shows that the ‘stage of disease’ is a significant risk factor for LC patient survival. After adjusting for all variables in the model, ‘stage of disease’ confirmed a significant risk factor of patient survival. Stage IV was associated with an 8.32–fold mortality risk compared to stages I and II (adjusted HR=8.32; 95%CI: 5.36 to 12.90), and stage III had a 6.35–fold mortality risk compared to stages I and II (adjusted HR=6.35; 95%CI: 4.05 to 9.95).

The results agree with a previous study wherein, compared to Stage 1, the respective mortality risk of Stage IIIB/IV, Stage IIIA, and Stage II was 5.34–fold (adjusted HR=5.34; 95%CI: 4.95 to 5.76), 2.71–fold (adjusted HR=2.71; 95%CI: 2.49 to 2.95), and 1.80–fold (adjusted HR=1.80; 95%CI: 1.65 to 1.97) (Sculier et al., 2008). In contrast, pulmonary large cell carcinoma is an infrequent neoplasm with a poor prognosis for which ‘stage of disease’ had a weak association with increased mortality risk. In that study, compared to stage I, the respective increased mortality risk of Stage II, III, and IV was 1.28–fold (adjusted HR=1.28; 95% CI: 1.09 to 1.51), 1.82 (adjusted HR=1.82; 95% CI: 1.59 to 2.10), and 3.45 (adjusted HR=3.45; 95% CI: 3.01to 4.02) (Xiaochuan et al., 2020). 

*Histologic type*

The current study showed that histology type was not a significant risk factor for LC survival. Compared to squamous cell carcinoma, the respective associated mortality risk for adenocarcinoma, small cell carcinoma, large cell carcinoma was 0.78–fold (adjusted HR=0.78; 95% CI: 0.63 to 0.96), 0.79–fold (adjusted HR=0.79; 95% CI: 0.50 to 1.22), and 1.01–fold (adjusted HR=1.01; 95% CI: 0.80 to 1.27). Compared to squamous cell carcinoma, the respective associated. 

Consistent with a previous study, adenocarcinoma and large cell carcinoma were associated with a 0.97-fold (adjusted HR=0.97; 95%CI: 0.68 to 1.37) and a 1.01-fold (adjusted HR=0.01; 95%CI: 0.61 to 1.78] mortality risk compared to squamous cell carcinoma (Srisam-ang et al., 2005). 

For mortality risk for NSCLC, adenocarcinoma and large cell was 0.96–fold (adjusted HR=0.96; 95% CI: 0.83 to 1.11), 0.95 (adjusted HR=0.95, 95%CI: 0.65 to 1.39) (Brzezniak et al., 2015).

*Chemotherapy*

The current study showed that chemotherapy afforded a significant advantaged vis-à-vis LC survival. After adjusting for all variables in the model, patients undergoing chemotherapy had improved survival. Chemotherapy was associated with a 0.48–fold mortality risk compared to non-chemotherapy (adjusted HR=0.48; 95%CI: 0.42 to 0.55). Our finding is consistent with prior studies. Chemotherapy alone was associated with a 0.55–fold mortality risk compared to no-treatment (adjusted HR=0.55; 95%CI: 0.54 to 0.56) (Lou et al., 2018). For advanced non-small cell lung cancer, chemotherapy alone was associated with a 0.38–fold mortality risk compared to no-treatment (adjusted HR=0.38; 95%CI: 0.37 to 0.39) and chemotherapy and surgery a 0.22–fold mortality risk compared to no-treatment (adjusted HR=0.22; 95%CI: 0.20 to 0.24) (David et al., 2016 ). 

*Advantages and Disadvantages of the study*

To our knowledge, the current study is the most up-to-date examination of LC survival factors post-diagnosis between 2013 and 2017, according to the Srinagarind Hospital-Based Cancer Registry. Based on available data, targeted therapy significantly improved OS in LC patients of all ages, all cells types (NSCLC and SCLC), but further confirmatory research using extensive prospective clinical trials is needed. Novel targeted systemic therapies and the appropriate selection of LC patient treatments based on tumor molecular phenotypes and histologies should also be reviewed. The limitations of our study are the relatively small number of patients receiving targeted therapy. 

In conclusion, the study confirmed that sex, stage of disease, histology, and chemotherapy are related to the survival of lung cancer patients. Primary prevention and screening for early detection are thus needed to improve survival. Factors affecting lung cancer survival in northeastern Thailand should focus on targeted therapy in any further investigations.

## Author Contribution Statement

WM is a principal investigator and provided project management supervision. SK and CJ provided advice about the study design and statistical analyses. CS provided and supervised the interviewers, and assisted in assessing data quality. AP is a physician and operated on patients with apparent Lung cancer and assisted in the final diagnoses of the cases. SK was involved in exploratory analysis and data quality. 
